# 4-Eth­oxy­anilinium chloride

**DOI:** 10.1107/S1600536810025973

**Published:** 2010-07-07

**Authors:** Xue-qun Fu

**Affiliations:** a Ordered Matter Science Research Center, Southeast University, Nanjing 210096, People’s Republic of China

## Abstract

The title compound, C_8_H_12_NO^+^·Cl^−^, consists of an almost planar protonated 4-eth­oxy­anilinium cation with the N atom showing the biggest deviation from the plane formed by all non-H atoms of the cation [0.066 (1) Å]. In the crystal, N—H⋯Cl hydrogen bonds link cations and anions into chains along the *a* axis. Additional C—H⋯π and π–π inter­actions [centroid–centroid distance = 4.873 (2) Å] stabilize the crystal structure.

## Related literature

For background to phase-transition materials, see: Li *et al.* (2008 ▶); Ye *et al.* (2009 ▶); Zhang *et al.* (2009 ▶). For similar structures, see: Fu (2009 ▶); Jiang *et al.* (1996 ▶); Zhao (2009 ▶).
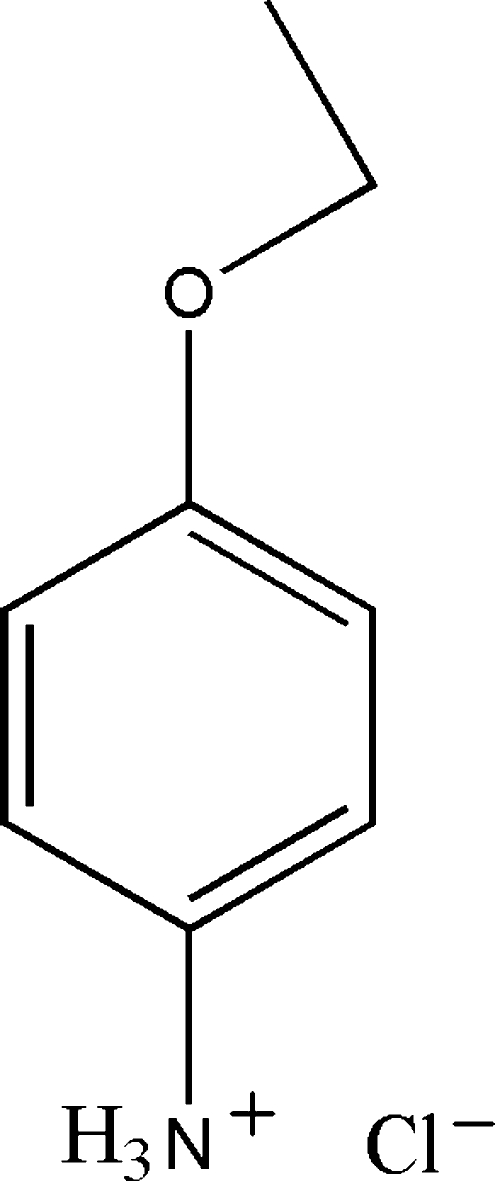


## Experimental

### 

#### Crystal data

C_8_H_12_NO^+^·Cl^−^
*M* *_r_* = 173.64
Orthorhombic, 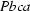

*a* = 11.422 (2) Å
*b* = 7.0890 (14) Å
*c* = 22.887 (5) Å
*V* = 1853.2 (6) Å^3^
*Z* = 8
Mo *K*α radiation
μ = 0.36 mm^−1^
*T* = 298 K
0.40 × 0.30 × 0.20 mm

#### Data collection

Rigaku SCXmini diffractometer
Absorption correction: multi-scan (*CrystalClear*; Rigaku, 2005 ▶) *T*_min_ = 0.879, *T*_max_ = 0.931
17046 measured reflections
2116 independent reflections
1655 reflections with *I* > 2σ(*I*)
*R*_int_ = 0.044

#### Refinement

*R*[*F* ^2^ > 2σ(*F* ^2^)] = 0.045
*wR*(*F* ^2^) = 0.106
*S* = 1.08
2116 reflections
112 parameters
H atoms treated by a mixture of independent and constrained refinement
Δρ_max_ = 0.27 e Å^−3^
Δρ_min_ = −0.23 e Å^−3^

### 

Data collection: *CrystalClear* (Rigaku, 2005 ▶); cell refinement: *CrystalClear*; data reduction: *CrystalClear*; program(s) used to solve structure: *SHELXS97* (Sheldrick, 2008 ▶); program(s) used to refine structure: *SHELXL97* (Sheldrick, 2008 ▶); molecular graphics: *SHELXTL* (Sheldrick, 2008 ▶); software used to prepare material for publication: *SHELXL97*.

## Supplementary Material

Crystal structure: contains datablocks I, global. DOI: 10.1107/S1600536810025973/im2216sup1.cif

Structure factors: contains datablocks I. DOI: 10.1107/S1600536810025973/im2216Isup2.hkl

Additional supplementary materials:  crystallographic information; 3D view; checkCIF report

## Figures and Tables

**Table 1:** Hydrogen-bond geometry (Å, °) *Cg*1 is the centroid of the C1–C6 ring.

*D*—H⋯*A*	*D*—H	H⋯*A*	*D*⋯*A*	*D*—H⋯*A*
N1—H1*D*⋯Cl1^i^	0.94 (2)	2.23 (3)	3.104 (2)	154 (2)
N1—H1*C*⋯Cl1^ii^	0.87 (3)	2.27 (3)	3.107 (2)	161 (2)
N1—H1*B*⋯Cl1	0.90 (3)	2.23 (3)	3.114 (2)	172 (2)
C4—H4*A*⋯*Cg*1^iii^	0.93	2.91	3.654 (2)	138
C7—H7*B*⋯*Cg*1^iv^	0.97	2.89	3.710 (2)	143

Symmetry codes: (i) 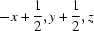; (ii) 
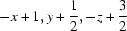
; (iii) 
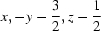
; (iv) 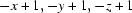.

**Fig. 1.**
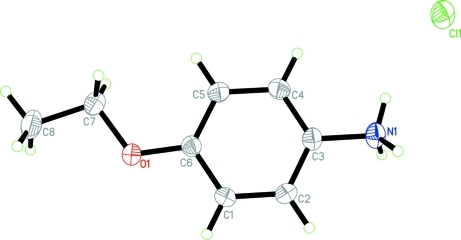
Molecular structure of the title compound, with the atomic numbering scheme. Displacement ellipsoids are drawn at the 30% probability level.

**Fig. 2.**
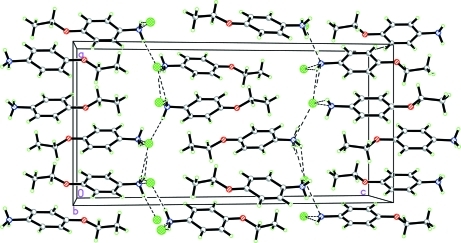
A view of the packing of the title compound, stacking along the b axis. Dashed lines indicate hydrogen bonds.

## supplementary crystallographic information

### Comment

The crystal structure of 4-ethoxyanilinium perchlorate as well as those of 2- and 4-methoxyanilinium chloride are known (Fu, 2009; Zhao, 2009; Jiang *et al.*, 1996). In this article, the crystal structure of (I) is presented.

The asymmetric unit of the title compound is built by an almost planar protonated 4-ethoxyanilinium cation and a Cl^-^ anion (Fig. 1). C—H···π interactions with a C4—H4A···*Cg*1 distance of 3.654 (2) Å and a C7—H7B···*Cg*1 distance of 3.710 (2) Å, respectively, as well as π-π packing interactions of adjacent benzene rings with a *Cg*1—*Cg*1 distance of 4.873 (2) Å, make a great contribution to the observed crystal structure (*Cg*1 is the centroid of benzene ring). Additional N—H···Cl hydrogen bonding with N—Cl distances of 3.104 (2) Å to 3.114 (2) Å (Table.1) link the cations and anions into chains along *a* axis (Fig.2).

### Experimental

Single crystals suitable for X-ray diffraction were obtained by slow evaporation at room temperature of an ethanolic solution of equimolar amounts of 4-ethoxyaniline and 6*M* hydrochloric acid.

Dielectric studies (capacitance and dielectric loss measurements) were performed using an automatic impedance TongHui2828 Analyzer on powder samples that were pressed into tablets on the surfaces of which a conducting carbon glue was deposited. Dielectric permittivity of the compound was tested to systematically to investigate the possibility of ferroelectric phase transitions (Li *et al.*, 2008, Ye *et al.*, 2009; Zhang *et al.*, 2009). Unfortunately, the temperature dependence of the relative permittivity at 1 MHz varied smoothly from 4.0 to 4.3 and there was no distinct anomaly observed from 93 K to 350 K (sublimation higher than 378 K) in the title compound, suggesting that this compound should not be a real ferroelectric or that no distinct phase transition occurred within the measured temperature range.

### Refinement

Positional parameters of all the H atoms for C atoms were calculated geometrically and were allowed to ride on the C atoms to which they are bonded, with *U*_iso_(H) = 1.2*U*_eq_(C). H atoms bonded to nitrogen atom were found in the difference maps and refined freely.

### Crystal data

**Table:**

C_8_H_12_NO^+^·Cl^−^	*F*(000) = 736
*M**_r_* = 173.64	*D*_x_ = 1.245 Mg m^−^^3^
Orthorhombic, *P**b**c**a*	Mo *K*α radiation, λ = 0.71073 Å
Hall symbol: -P 2ac 2ab	Cell parameters from 7266 reflections
*a* = 11.422 (2) Å	θ = 3.0–27.7°
*b* = 7.0890 (14) Å	µ = 0.36 mm^−^^1^
*c* = 22.887 (5) Å	*T* = 298 K
*V* = 1853.2 (6) Å^3^	Prism, colourless
*Z* = 8	0.40 × 0.30 × 0.20 mm

### Data collection

**Table:**

Rigaku SCXmini diffractometer	2116 independent reflections
Radiation source: fine-focus sealed tube	1655 reflections with *I* > 2σ(*I*)
graphite	*R*_int_ = 0.044
Detector resolution: 13.6612 pixels mm^-1^	θ_max_ = 27.5°, θ_min_ = 3.5°
ω scans	*h* = −14→14
Absorption correction: multi-scan (*CrystalClear*; Rigaku, 2005)	*k* = −9→9
*T*_min_ = 0.879, *T*_max_ = 0.931	*l* = −29→29
17046 measured reflections	

### Refinement

**Table:**

Refinement on *F*^2^	Primary atom site location: structure-invariant direct methods
Least-squares matrix: full	Secondary atom site location: difference Fourier map
*R*[*F*^2^ > 2σ(*F*^2^)] = 0.045	Hydrogen site location: inferred from neighbouring sites
*wR*(*F*^2^) = 0.106	H atoms treated by a mixture of independent and constrained refinement
*S* = 1.08	*w* = 1/[σ^2^(*F*_o_^2^) + (0.0383*P*)^2^ + 0.7382*P*] where *P* = (*F*_o_^2^ + 2*F*_c_^2^)/3
2116 reflections	(Δ/σ)_max_ < 0.001
112 parameters	Δρ_max_ = 0.27 e Å^−^^3^
0 restraints	Δρ_min_ = −0.23 e Å^−^^3^

### Special details

**Table:**

Geometry. All e.s.d.'s (except the e.s.d. in the dihedral angle between two l.s. planes) are estimated using the full covariance matrix. The cell e.s.d.'s are taken into account individually in the estimation of e.s.d.'s in distances, angles and torsion angles; correlations between e.s.d.'s in cell parameters are only used when they are defined by crystal symmetry. An approximate (isotropic) treatment of cell e.s.d.'s is used for estimating e.s.d.'s involving l.s. planes.
Refinement. Refinement of *F*^2^ against ALL reflections. The weighted *R*-factor *wR* and goodness of fit *S* are based on *F*^2^, conventional *R*-factors *R* are based on *F*, with *F* set to zero for negative *F*^2^. The threshold expression of *F*^2^ > σ(*F*^2^) is used only for calculating *R*-factors(gt) *etc*. and is not relevant to the choice of reflections for refinement. *R*-factors based on *F*^2^ are statistically about twice as large as those based on *F*, and *R*- factors based on ALL data will be even larger.

### Fractional atomic coordinates and isotropic or equivalent isotropic displacement parameters (Å^2^)

**Table:**

	*x*	*y*	*z*	*U*_iso_*/*U*_eq_	
Cl1	0.36897 (4)	0.14153 (8)	0.73645 (2)	0.0565 (2)	
C3	0.39597 (14)	0.6120 (3)	0.64180 (8)	0.0382 (4)	
O1	0.39659 (12)	0.76724 (19)	0.46863 (5)	0.0495 (4)	
C6	0.39549 (15)	0.7056 (3)	0.52510 (8)	0.0384 (4)	
N1	0.39087 (15)	0.5668 (3)	0.70418 (7)	0.0456 (4)	
H1D	0.323 (2)	0.619 (3)	0.7215 (10)	0.072 (7)*	
H1C	0.451 (2)	0.614 (4)	0.7224 (11)	0.081 (8)*	
H1B	0.387 (2)	0.442 (5)	0.7096 (12)	0.084 (9)*	
C7	0.34271 (19)	0.6523 (3)	0.42485 (8)	0.0509 (5)	
H7A	0.2601	0.6365	0.4333	0.061*	
H7B	0.3791	0.5287	0.4240	0.061*	
C5	0.34210 (18)	0.5419 (3)	0.54364 (9)	0.0511 (5)	
H5A	0.3060	0.4626	0.5168	0.061*	
C4	0.34242 (17)	0.4958 (3)	0.60251 (9)	0.0507 (5)	
H4A	0.3061	0.3858	0.6152	0.061*	
C1	0.45119 (17)	0.8200 (3)	0.56533 (8)	0.0468 (5)	
H1A	0.4890	0.9289	0.5528	0.056*	
C8	0.3583 (2)	0.7490 (4)	0.36708 (9)	0.0651 (6)	
H8A	0.3226	0.6748	0.3369	0.098*	
H8B	0.4403	0.7631	0.3590	0.098*	
H8C	0.3220	0.8711	0.3684	0.098*	
C2	0.45113 (17)	0.7739 (3)	0.62369 (8)	0.0456 (5)	
H2A	0.4882	0.8517	0.6507	0.055*	

### Atomic displacement parameters (Å^2^)

**Table:**

	*U*^11^	*U*^22^	*U*^33^	*U*^12^	*U*^13^	*U*^23^
Cl1	0.0385 (3)	0.0715 (4)	0.0596 (3)	−0.0036 (2)	−0.0107 (2)	0.0117 (3)
C3	0.0278 (8)	0.0501 (11)	0.0366 (9)	0.0017 (7)	−0.0005 (7)	0.0041 (8)
O1	0.0676 (9)	0.0474 (8)	0.0334 (7)	−0.0125 (7)	−0.0020 (6)	0.0020 (6)
C6	0.0387 (9)	0.0406 (10)	0.0359 (9)	−0.0017 (8)	0.0015 (7)	0.0015 (8)
N1	0.0329 (9)	0.0653 (13)	0.0387 (9)	0.0010 (8)	−0.0008 (7)	0.0082 (9)
C7	0.0576 (12)	0.0565 (12)	0.0387 (10)	−0.0041 (10)	−0.0017 (8)	−0.0069 (9)
C5	0.0571 (12)	0.0524 (12)	0.0438 (11)	−0.0209 (10)	−0.0067 (9)	0.0004 (9)
C4	0.0510 (11)	0.0532 (12)	0.0477 (11)	−0.0204 (9)	−0.0022 (8)	0.0079 (10)
C1	0.0583 (12)	0.0393 (10)	0.0428 (10)	−0.0122 (9)	0.0003 (9)	0.0029 (8)
C8	0.0884 (17)	0.0686 (15)	0.0382 (11)	0.0068 (13)	−0.0028 (10)	−0.0032 (11)
C2	0.0511 (11)	0.0438 (11)	0.0418 (10)	−0.0087 (9)	−0.0053 (8)	−0.0045 (8)

### Geometric parameters (Å, °)

**Table:**

C3—C4	1.364 (3)	C7—H7A	0.9700
C3—C2	1.373 (3)	C7—H7B	0.9700
C3—N1	1.464 (2)	C5—C4	1.386 (3)
O1—C6	1.364 (2)	C5—H5A	0.9300
O1—C7	1.431 (2)	C4—H4A	0.9300
C6—C5	1.378 (3)	C1—C2	1.375 (3)
C6—C1	1.382 (3)	C1—H1A	0.9300
N1—H1D	0.94 (2)	C8—H8A	0.9600
N1—H1C	0.87 (3)	C8—H8B	0.9600
N1—H1B	0.90 (3)	C8—H8C	0.9600
C7—C8	1.500 (3)	C2—H2A	0.9300
			
C4—C3—C2	120.75 (17)	C6—C5—C4	119.76 (18)
C4—C3—N1	119.51 (18)	C6—C5—H5A	120.1
C2—C3—N1	119.70 (17)	C4—C5—H5A	120.1
C6—O1—C7	118.49 (15)	C3—C4—C5	119.98 (18)
O1—C6—C5	124.45 (17)	C3—C4—H4A	120.0
O1—C6—C1	116.03 (16)	C5—C4—H4A	120.0
C5—C6—C1	119.51 (17)	C2—C1—C6	120.50 (17)
C3—N1—H1D	110.9 (14)	C2—C1—H1A	119.7
C3—N1—H1C	110.6 (17)	C6—C1—H1A	119.7
H1D—N1—H1C	107 (2)	C7—C8—H8A	109.5
C3—N1—H1B	110.7 (18)	C7—C8—H8B	109.5
H1D—N1—H1B	107 (2)	H8A—C8—H8B	109.5
H1C—N1—H1B	111 (2)	C7—C8—H8C	109.5
O1—C7—C8	107.79 (18)	H8A—C8—H8C	109.5
O1—C7—H7A	110.1	H8B—C8—H8C	109.5
C8—C7—H7A	110.1	C3—C2—C1	119.47 (17)
O1—C7—H7B	110.1	C3—C2—H2A	120.3
C8—C7—H7B	110.1	C1—C2—H2A	120.3
H7A—C7—H7B	108.5		
			
C7—O1—C6—C5	−2.4 (3)	C6—C5—C4—C3	−0.3 (3)
C7—O1—C6—C1	178.51 (17)	O1—C6—C1—C2	177.53 (18)
C6—O1—C7—C8	−178.74 (17)	C5—C6—C1—C2	−1.6 (3)
O1—C6—C5—C4	−177.57 (19)	C4—C3—C2—C1	0.7 (3)
C1—C6—C5—C4	1.5 (3)	N1—C3—C2—C1	−177.14 (18)
C2—C3—C4—C5	−0.8 (3)	C6—C1—C2—C3	0.5 (3)
N1—C3—C4—C5	177.04 (18)		

### Hydrogen-bond geometry (Å, °)

**Table:**

Cg1 is the centroid of the C1–C6 ring.

**Table:**

*D*—H···*A*	*D*—H	H···*A*	*D*···*A*	*D*—H···*A*
N1—H1D···Cl1^i^	0.94 (2)	2.23 (3)	3.104 (2)	154 (2)
N1—H1C···Cl1^ii^	0.87 (3)	2.27 (3)	3.107 (2)	161 (2)
N1—H1B···Cl1	0.90 (3)	2.23 (3)	3.114 (2)	172 (2)
C4—H4A···Cg1^iii^	0.93	2.91	3.654 (2)	138
C7—H7B···Cg1^iv^	0.97	2.89	3.710 (2)	143

Symmetry codes: (i) −*x*+1/2, *y*+1/2, *z*; (ii) −*x*+1, *y*+1/2, −*z*+3/2; (iii) *x*, −*y*−3/2, *z*−1/2; (iv) −*x*+1, −*y*+1, −*z*+1.

## References

[bb1] Fu, X. (2009). *Acta Cryst.* E**65**, o2345. 10.1107/S1600536809035041 PMC2970473 21577815

[bb2] Jiang, Z.-T., Liesegang, J., James, B. D., Skelton, B. W. & White, A. H. (1996). *J. Phys. Chem. Solids*, **57**, 397–404.

[bb3] Li, X. Z., Qu, Z. R. & Xiong, R. G. (2008). *Chin. J. Chem.* **11**, 1959–1962.

[bb4] Rigaku (2005). *CrystalClear* Rigaku Corporation, Tokyo, Japan.

[bb5] Sheldrick, G. M. (2008). *Acta Cryst.* A**64**, 112–122. 10.1107/S0108767307043930 18156677

[bb6] Ye, H. Y., Fu, D. W., Zhang, Y., Zhang, W., Xiong, R. G. & Huang, S. P. (2009). *J. Am. Chem. Soc.* **131**, 42–43. 10.1021/ja808331g 19128170

[bb7] Zhang, W., Chen, L. Z., Xiong, R. G., Nakamura, T. & Huang, S. D. (2009). *J. Am. Chem. Soc.* **131**, 12544–12545. 10.1021/ja905399x 19685869

[bb8] Zhao, M. M. (2009). *Acta Cryst.* E**65**, o2378. 10.1107/S1600536809035429 PMC2970478 21577843

